# Mechanisms of Itch in Atopic Dermatitis

**DOI:** 10.14789/ejmj.JMJ24-0036-R

**Published:** 2025-01-30

**Authors:** YAYOI KAMATA, MITSUTOSHI TOMINAGA, KENJI TAKAMORI

**Affiliations:** 1 Juntendo Itch Research Center (JIRC), Institute for Environmental and Gender-Specific Medicine, Juntendo University Graduate School of Medicine, Chiba, Japan; 1 Juntendo Itch Research Center (JIRC), Institute for Environmental and Gender-Specific Medicine, Juntendo University Graduate School of Medicine, Chiba, Japan; 2 Department of Dermatology, Juntendo University Urayasu Hospital, Chiba, Japan; 2 Department of Dermatology, Juntendo University Urayasu Hospital, Chiba, Japan

**Keywords:** atopic dermatitis, intraepidermal nerve fibers, itch, neuroimmune communication

## Abstract

Atopic dermatitis (AD) is a common inflammatory skin disease characterized by recurrent eczematous lesions and intense itch. The pathological mechanism of AD involves a complex interaction between skin barrier dysfunction and a predominantly T helper (Th) 2-skewed immune dysregulation. The dysfunctional skin barrier in AD enhances antigen penetration, exacerbating allergic reactions. Scratching further damages the skin barrier, worsens dryness and increases the release of pro-inflammatory mediators, perpetuating the itch-scratch cycle. Breaking this cycle with appropriate treatments is vital. Th2 cells secrete interleukin (IL)-4, IL-13 and IL-31 which play keys roles in AD pathogenesis. IL-31 directly induces pruritus, while IL-4 and IL-13 enhance itching. An increased density of intraepidermal nerve fibers has been observed in AD lesions in a disease-state-dependent manner. In normal skin, both semaphorin 3A (Sema3A; a nerve repulsion factor) and nerve growth factor (NGF; a nerve elongation factor) are expressed. However, in AD lesions, Sema3A expression decreases while NGF expression increases. These findings suggest that epidermal nerve density is regulated by a fine balance between Sema3A and NGF, with Sema3A playing a key role in itch sensitivity in AD. In healthy skin, Sema3A is produced during the early-stage of differentiation of keratinocytes and moves into the upper epidermis. The levels of Sema3A and the density of epidermal nerve fibers may vary depending on the disease state of AD. Our future research will focus on the regulatory mechanisms of Sema3A in skin, and potential clinical applications.

## Introduction

Atopic dermatitis (AD) is one of the most common inflammatory skin diseases, with a lifetime prevalence of 15 to 20% in developed countries, which is much higher than that of other atopic diseases^1)^. It is characterized by intractable itching and recurrent eczematous lesions. The onset of AD often precedes other allergic diseases throughout life, typically progressing in a pattern known as the “atopic march,” which involves food allergies, followed by asthma, and eventually allergic rhinitis^2)^. The causes of AD are complex and multifactorial, with skin barrier dysfunction and type 2 inflammation being generally recognized as key contributors to its pathogenesis. AD is driven by a “trinity” of factors: skin barrier dysfunction, inflammation/dermatitis, and itching, all of which play roles in the development and progression of the disease^3)^. Understanding the complex interplay among these factors is crucial for developing effective treatments and managing symptoms to maintain patients’ quality of life (QOL). In this review, we discuss the mechanisms underlying itch in AD and summarize the regulation of intraepidermal nerve fibers as a cause of itching.

## Itch-scratch cycle

The skin barrier primarily depends on the stratum corneum and tight junctions to protect the skin by preventing excess water loss and blocking allergen entry. Filaggrin (FLG) plays a crucial role in forming the stratum corneum by facilitating keratinization, where living cells are transformed into hardened, dead cells called corneocytes^4)^. FLG monomers are eventually degraded by proteases into free amino acids, known as natural moisturizing factors (NMF)^3-6)^. The integrity of the skin barrier is essential for maintaining skin homeostasis, but it can be compromised by genetic, environmental, and other factors. FLG gene mutations, for example, have been identified as the cause of ichthyosis vulgaris, a condition often associated with AD^7)^. Additional studies have confirmed this association and identified other loss-of-function mutations in the FLG gene that predispose individuals to AD. These mutations are present in 7 to 10% of the white European population^7, 8)^. Environmental factors, such as scratching, exogenous proteases of house dust mites, and *Staphylococcus aureus*, can also disrupt the skin barrier^5)^. Other factors, such as decreased ceramide levels, further contribute to skin barrier dysfunction^9)^. When the epidermal barrier is compromised, allergens can penetrate the skin more easily, interacting with local antigen-presenting and immune effector cells^5)^. This triggers the release of alarmins, such as thymic stromal lymphopoietin (TSLP) and interleukin (IL)-33, which in turn activate type 2 immune responses^1)^. Activated T-helper (Th) 2 cells release IL-4 and IL-13, promoting IgE class switching in B cells and production of antigen-specific IgE^1, 10)^. IL-4 and IL-13 also induce chemokine production in keratinocytes, which accelerates the infiltration of inflammatory cells into the skin^11)^.

**Figure 1:**
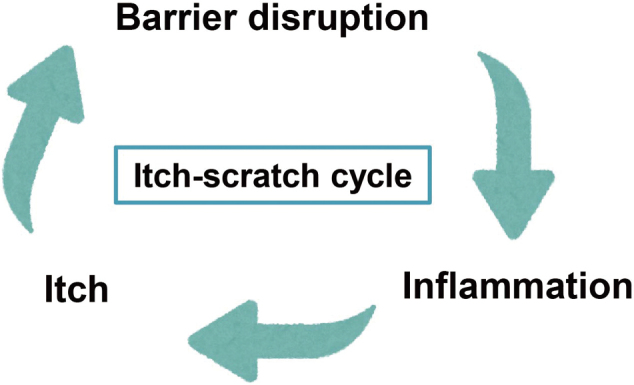
The itch-scratch cycle In atopic dermatitis, a dysfunctional skin barrier allows increased antigen penetration, leading to aggravated allergic reactions. The loss of moisture and pathogen infiltration worsen dryness and inflammation, inducing an urge to scratch. Scratching further damages the skin barrier, increases dryness, and enhances the release of pro-inflammatory mediators, perpetuating a vicious itch-scratch cycle.

Pruritus is an unpleasant sensation that serves as a self-protective mechanism, helping the body avoid harm from external irritants^12, 13)^. In AD, pruritus is characterized by itch hypersensitivity, including alloknesis and hyperkinesis^12, 13)^. Alloknesis refers to innocuous tactile stimuli, such as light touches from clothing, that evoke itching, while hyperkinesis refers to an exaggerated itch response to normal itchy stimuli. Although histamine is a well-known itch-inducing substance, antihistamines (histamine H1-receptor blockers) are not fully effective in treating itch in AD^14)^. This intractable itch is a clinical challenge that significantly reduces patients’ QOL. As shown in Figure 1, the dysfunctional barrier in AD leads to enhanced antigen penetration, exacerbating allergic reactions. Scratching further damages the skin barrier, worsens dryness, and increases the release of pro-inflammatory mediator, perpetuating the itch-scratch cycle. Breaking this cycle with appropriate treatments is essential to manage itch effectively.

## Cutaneous barrier disruption and itch sensitivity

In human skin, primary sensory afferents can be categorized by their degree of myelination, conduction velocity, axonal diameter, and nerve ending type^15)^. Itch is primarily conducted by slow-conducting, unmyelinated C fibers. Endogenous pruritogenic factors produced by epidermal keratinocytes and immune cells, such as mast cells and T cells, act on and excite sensory nerve fibers through their corresponding receptors and channels (Table 1). When sensory nerves are excited, they release itch transmitters like natriuretic peptide B, gastrin- releasing peptide, glutamate, and neurokinin B into the dorsal horn of the spinal cord. These neurotransmitters stimulate neurons in the spinal dorsal horn, which then transmit itch signals to the brain via the spinal cord-thalamic tract or the spinal cord- parabrachial nucleus tract^16, 17)^.

**Table 1:** Itch mediators and receptors

Category	Itch mediators	Receptor
Amines	Histamine Serotonin (5-HT)	H_1_R, H_4_R, TRPV1 5-HT_2A_, 5-HT_7_, TRPV4
Proteases	SLIGRL Tryptase Mucunain Cathepsin S	Protease-activated receptor (PAR) -2, MrgprC11 PAR-2 PAR-2, PAR-4 PAR-2, PAR-4, MrgprC11
Neuropeptides	Substance P Endothelin (ET)-1	NK1 receptor, MrgprA1 ET_A_ receptor
Lipids	Platelet-activating factor (PAF) Lysophosphatidic acid (LPA) Leukotriene B4 (LTB4)	PAF receptor LPA_5_, TRPA1 BLT1 receptor
Cytokines	IL-31 TSLP CXCL10 (IP-10)	IL-31 RA, OSMR, TRPV1, TRPA1 TSLP receptor, TRPA1 CXCR3
Mrgpr agonist	Chloroquine BAM8-22 β-alanine	MrgprX1(human), MrgprA3(mouse), TRPA1 MrgprX1(human), MrgprC11(mouse), TRPA1 MrgprD
Others	Bile acid Oxidative stress	TGR5, TRPA1 TRPA1

**Figure 2:**
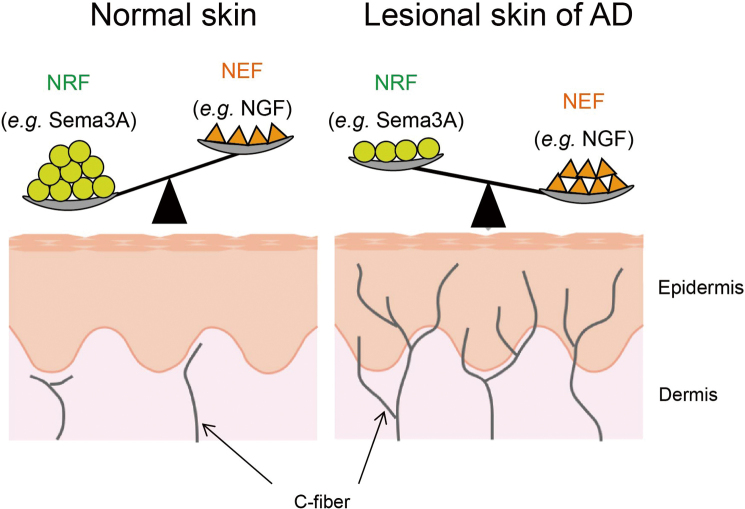
Regulation of axonal guidance molecule expression in skin In healthy skin, the levels of nerve repulsion factor (NRF), such as semaphorin 3A (Sema3A) are more dominant than nerve elongation factors (NEF), like nerve growth factor (NGF). This balance prevents nerve fibers from easily penetrating the epidermis. In contrast, in the lesional skin of atopic dermatitis (AD), NEF are more dominant than NRF, allowing nerve fibers to penetrate the epidermis.

In healthy skin, most cutaneous nerve fibers terminate at the dermoepidermal junction (Figure 2)^18)^. However, in barrier-disrupted skin, such as the lesional skin of AD or dry skin, an increased density of intraepidermal nerves has been observed, which contributes to itch sensitization. This hyperinnervation is partly responsible for increased itch sensitivity. Since this type of pruritus does not involve histamine, H_1_-antihistamines are ineffective for treating it. Additionally, intraepidermal nerve fiber terminals are usually trimmed at the tight junctions of the stratum granulosum (SG) 2 layer^19)^. In AD model mice, however, this trimming process is disrupted, leading to nerve fibers penetrating through and protruding into the outer skin layers, resulting in abnormal sensory nerve activation. Primary sensory nerves in the skin are responsible for transmitting itch signals. Recent single-cell RNA sequencing has classified the sensory neuron system into five neurofilament clusters, two clusters of peptidergic nociceptors, one tyrosine hydroxylase containing cluster, and three clusters of non-peptidergic (NP) nociceptors^20)^. Neurons in the NP1, NP2, and NP3 clusters express receptor genes for itch mediators^20, 21)^. NP1 neurons strongly express Mas- related G protein coupled receptor (Mrgpr) D, while NP2 neurons express MrgprA3, MrgprC11, and the histamine H_1_ receptor. NP3 neurons express the IL-31 receptor, serotonin receptors, histamine H_1_ receptor, and leukotriene C4 receptors. All three clusters, NP1, NP2, and NP3, commonly express IL-4 and IL-13 receptors^20, 21)^. However, it remains unclear which subsets of neurons are increased in AD lesions.

Periostin, an extracellular matrix and matricellular protein, is emerging as a significant player in itch sensation^22-24)^. In human inflamed skin, such as in AD, periostin is deposited throughout the dermis. It acts on keratinocytes by activating NF-κB, leading to the production of pro-inflammatory cytokines, including TSLP^25)^. Periostin also binds to αvβ3 integrin and directly stimulates nerve fibers, resulting in itch sensation^26)^.

## Neuroimmune communication

In the non-lesional skin of AD, keratinocytes secrete cytokines known as alarmins due to the disrupted skin barrier. These cytokines activate type 2 innate lymphoid cells, which in turn induce type 2 inflammation through IL-5 and IL-13. Th2 cells secrete IL-4, IL-13 and IL-31, playing an important role in the pathogenesis of AD^10)^. In particular, IL-31 directly induces pruritus and increases in the lesional skin and serum of AD patients^27-29)^. NP3 neurons express IL-31R, suggesting a significant contribution to itch^20, 21)^. IL-31 is primarily produced by Th2 cells, mast cells, and macrophages^30)^. Nemolizumab, a humanized IL-31RA monoclonal antibody marketed as Mitchga, was approved in Japan in March 2022^31)^. In IL-31 transgenic mice, the number of PGP9.5^+^ nerve fibers and the thickness of epidermis increased in AD-like lesions^32)^. IL-31RA is expressed on small-diameter neurons, and IL-31 selectively promotes nerve fiber extension in these neurons^32, 33)^. Collectively, IL-31 is not only a mediator of itch but also induces neurite elongation.

Some cytokines act as modulators rather than direct itch mediators, enhancing or attenuating itch. For instance, cytokines such as IL-4, IL-13, IL-33, and oncostatin M enhance itch^34-37)^. IL-4, released from Th2 cells, binds to IL-4 receptors and is thought to induce itch and inflammation via the JAK-STAT pathway, contributing to the pathogenesis of AD^38)^. Although IL-4 does not directly induce itch, it enhances histamine-induced itch^34)^. Intradermal co-injection of a low dose of histamine with IL-4 significantly increased scratching behavior in mice compared to histamine alone^34)^. In dry or non-lesional skin of AD, IL-33 is secreted by keratinocytes in barrier-disrupted skin, inducing itch sensitization^16)^. In the lesional skin of AD, Th2 cytokines (IL-4 and IL-13) play a key role in itch sensitization^16, 36)^. Currently, several biologics targeting cytokine signaling, including dupilumab, nemolizumab, tralokinumab, and lebrikizumab are approved for use in Japan^39)^.

## Regulatory mechanisms of intraepidermal nerve fibers

In the lesional skin of AD, numerous nerve fibers penetrate the epidermis. Nerve elongation factors (NEF), such as nerve growth factor (NGF), promote neurite outgrowth, while nerve repulsion factors (NRF), like semaphorin 3A (Sema3A), inhibit it. In normal skin, NRF levels are more dominant than NEF, maintaining a balance that limits nerve fiber penetration into the epidermis. However, in the lesional skin of AD, NEF levels become dominant over NRF, allowing nerve fibers to more easily penetrate the epidermis. Both Sema3A and NGF are expressed in normal skin, but in AD lesions, Sema3A expression decreases while NGF expression increases^40)^. These findings suggest that epidermal nerve density is regulated by a delicate balance between NEF and NRF^16)^ (Figure 2). Previously, we developed an ointment containing recombinant Sema3A protein, which was applied to the lesional skin of AD model NC/Nga mice^41)^. Sema3A ointment significantly inhibited epidermal hyperinnervation and reduced scratching behavior. It also improved barrier function and dermatitis in the AD model mice compared to controls^41)^. However, there are challenges with clinical application. Protein-based drugs tend to be unstable and have high production costs. Thus, we proposed that activators of endogenous Sema3A expression might offer a promising treatment for intractable itch in AD.

## Regulatory mechanisms of Sema3A in normal human epidermal keratinocytes

**Figure 3:**
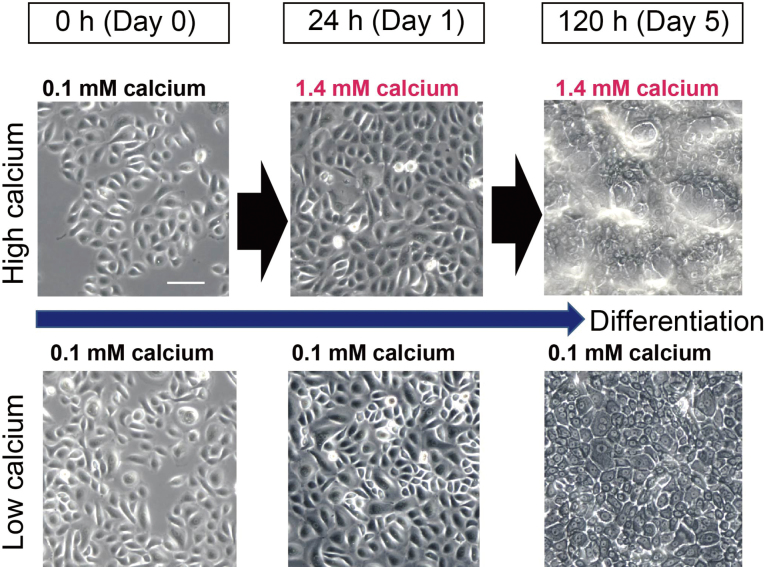
Cell morphology of normal human epidermal keratinocytes (NHEK) during calcium-induced differentiation Phase-contrast images show NHEK cultured in medium containing 0.1 mM or 1.4 mM calcium. Microscopic images were taken at each time point after medium replacement. Scale bar: 100 μm.

Calcium is a major regulator of keratinocyte differentiation both *in vivo* and *in vitro*^42, 43)^. It forms a gradient within the epidermis, with the highest concentration in the granular layer^43)^. However, the acute disruption of the epidermal permeability barrier causes a loss of this calcium gradient^43)^. Normal human epidermal keratinocytes (NHEK) undergo differentiation in the presence of high calcium levels (Figure 3). *Sema3A* mRNA levels were transiently increased in high calcium-stimulated NHEK, but markedly decreased in terminally differentiated NHEK. Using *in situ* hybridization, *Sema3A* mRNA was primarily detected in keratin 14-positive keratinocytes in the stratum basale and lower stratum spinosum. Furthermore, we constructed a human *Sema3A* promoter assay system and analyzed *Sema3A* promoter activity in NHEK. Site-directed mutagenesis of the activator protein (AP)-1 binding site significantly reduced *Sema3A* promoter activity compared to the intact plasmid. AP-1 is a basic-leucine zipper transcription factor^44)^ that forms multiple dimer pairs, such as Jun and Fos. AP-1 complexes are key regulators of keratinocyte survival and differentiation, and they are important downstream targets of mitogen activated protein kinase (MAPK) signaling^44-46)^. After stimulation with high calcium, JunB and Fra-2 were found to directly interact with the transcription factor-binding site in the proximal promoter region of *Sema3A*, as confirmed by chromatin immunoprecipitate assays. *Sema3A* expression increased when JunB and Fra-2 were co-expressed in the presence of 0.1 or 1.4 mM calcium, whereas overexpression of JunB or Fra-2 alone had no effect on *Sema3A* expression. Additionally, other combinations of Jun and Fos increased *Sema3A* mRNA expression. The MAPK-MEK/ERK1/2 pathway is involved in the differentiation of keratinocytes both in vivo and in vitro. The calcium-mediated transient up-regulation of *Sema3A* expression was significantly suppressed by the MEK1/2 inhibitor (PD98059) and the AP-1 inhibitor (T-5224). Taken together, we found that calcium-mediated transient upregulation of *Sema3A* in NHEK is dependent on the MEK/ERK and AP-1 signaling pathways^47)^. In healthy skin, *Sema3A* is produced during the early stages of keratinocyte differentiation and may subsequently move into the upper epidermis.

## Why Sema3A expression decreases in AD lesions

To investigate why Sema3A expression decreases in AD lesions, we conducted several analyses. Disruption of the epidermal barrier leads to the loss of the calcium gradient^43)^, which may downregulate Sema3A expression in the epidermis. We hypothesized that inflammation and skin barrier defects are the primary causes of decreased Sema3A expression in AD. In a murine model, IL-4 has been reported to reduce Sema3A expression in the skin^48)^. However, in NHEK, Th2 cytokines, such as IL-4 and IL-13, actually increased *Sema3A* mRNA expression. We also tested *Sema3A* gene expression in cytokine-stimulated reconstructed human epidermis (RHE) models, where IL-4 and IL-13 similarly increased *Sema3A* expression consistent with the results from monolayer cultures (unpublished data).

Next, we performed a barrier disruption experiment using detergent on the RHE model. Applying 0.2% Triton X-100 to the stratum corneum disrupted the epidermal barrier, causing transepidermal water loss to increase for 2 to 6 h before gradually decreasing over time (unpublished data). This suggested that barrier function deteriorated temporarily before recovery. *Sema3A* expression was transiently suppressed immediately after barrier disruption, but markedly increased after 24 and 48 h. In contrast, *NGF* expression showed an initial increase initially, but decreased after 24 and 48 h. These changes may reflect compensatory responses to the recovery of barrier function. Similarly, in an acetone-treated dry skin mouse model, barrier disruption increased nerve fiber density in the epidermis^49)^. Collectively, these findings suggest that in humans, cytokines alone may not directly downregulate Sema3A in AD lesions. Instead, barrier disruption likely plays a key role in Sema3A downregulation in AD.

## Development of Sema3A expression inducers

Drug repositioning, a strategy for discovering new uses for approved or investigational drugs, has led to identifying Sema3A expression inducers. For example, thalidomide, initially developed for morning sickness, is now used to treat multiple myeloma^50)^. Through drug repositioning, we screened the Prestwick Chemical Library aiming for Sema3A expression inducers. Among the 16 identified compounds, we focused on the antiparasitic drug parbendazole, which significantly induced Sema3A expression and secretion of NHEK (Unpublished data). Similar effects were observed with related antiparasitic drugs, fenbendazole and albendazole. These drugs also significantly suppressed *NGF* mRNA expression in NHEK.

Other compounds that induce Sema3A expression include the antimicrobial peptide LL-37, a member of the cathelicidin family^51)^, which we found to promote Sema3A expression^52)^. Additionally, extracts from *Scutellaria baicalensis* root, used in traditional herbal medicines, have been shown to promote Sema3A expression, with baicalin and baicalein identified as the active compounds^53)^.

## Conclusions

In AD lesions, many nerve fibers penetrate the epidermis. However, some studies suggest that while nerve fibers penetrate normal skin, their density decreases in AD lesions^54, 55)^. We hypothesize that changes in epidermal nerve fiber density may depend on the disease state of AD. Since IL-4 and IL-13 did not decrease Sema3A expression, it is believed that axon guidance molecule regulation involves not only cytokines but also complex mechanisms, including barrier destruction. Sema3A is a key molecule in preventing itch sensitivity in AD lesions. In the future, we aim to establish methods to regulate Sema3A expression in skin, and clinical applications.

## Funding

This work was partly supported by JSPS KAKENHI (22K08412, 19K08756, 16K19739), Lydia O’leary Memorial Pias Dermatological Foundation (2020), Mandom International Research Grant (2022) and Hoyu Research Foundation (2024).

## Author contributions

YK was a major contributor in writing the manuscript. MT and KT contributed to reviewing and editing the manuscript.

## Conflicts of interest statement

The authors declare that there are no conflicts of interest.
